# Normal pressure hydrocephalus – why treatment is often delayed or not even initiated

**DOI:** 10.1186/2045-8118-12-S1-P23

**Published:** 2015-09-18

**Authors:** Uwe Kehler

**Affiliations:** 1 Asklepios Hospital Hamburg Altona, Germany

## 

Normal pressure hydrocephalus is a common disease in elderly people and treatment is beneficent. It is also well known, that delayed treatment of NPH shows worth results than early treatment. However, many patients are sent with enormous delay and or not even sent to hydrocephalus specialists. The aim of this work is to search for the reasons and discuss improvements.

## Methods

The reasons for delayed treatment of NPH in patients who were treated finally in our department are summarized. Only reasons which occurred twice are mentioned not to overestimate too exceptional cases. Only cases were included who improved after shunt surgery.

## Results

The reasons for delayed NPH treatment could be identified: 1: Spinal tap testing did not show clear improvement, although patient felt substantial improvement. 2: Examination of spinal tap test was done at wrong time, patient improved substantially after demission of the hospital. 3: Radiologist misdiagnosed hydrocephalus (typical wrong diagnosis: brain atrophy and/or cerebral micro-angiopathy), 4: Patient was considered to be too old for shunt surgery. 5: General physician and/or neurologist considered surgery too risky (without explaining the patient the progressive natural history of the disease). 6. NPH was not suspected by the general physician and/or neurologist or NPH was misdiagnosed as Alzheimer's disease and Parkinson's disease.

## Discussion

Unawareness of NPH, general physician's and neurologist's fear of shunt complications, thoughtless radiological diagnosis of brain atrophy with excluding hydrocephalus are some reasons why patients are sent delayed to hydrocephalus specialists. Medical education and information has to be improved that NPH patients can get the benefit of treatment as early as possible.
